# LncRNA ZFAS1 promotes pancreatic adenocarcinoma metastasis via the RHOA/ROCK2 pathway by sponging miR-3924

**DOI:** 10.1186/s12935-020-01322-8

**Published:** 2020-06-16

**Authors:** Jinyang Liu, Yaqin Zhu, Chunlin Ge

**Affiliations:** 1 Department of Hepatobiliary and Pancreatic Surgery, Hunnan Division of The First Affiliated Hospital of China Medical University, Liaoning, China; 2 Key Laboratory of Medical Cell Biology, Ministry of Education of the PRC Affiliation, Liaoning, China; 3 School of Life Science, China Medical University, Liaoning, China

**Keywords:** PAAD, Long non-coding RNA, ZFAS1, miR-3924, ROCK2

## Abstract

**Background:**

The mortality and morbidity rates of pancreatic adenocarcinoma have been increasing over the past two decades, and an understanding of the mechanisms underlying pancreatic adenocarcinoma progression is urgently needed. The long non-coding RNA ZFAS1 has been demonstrated to be an oncogene in some cancers, but its function and mechanism in pancreatic adenocarcinoma remain unclear.

**Methods:**

The ZFAS1 expression level in pancreatic adenocarcinoma was predicted by bioinformatic analysis, and the expression level of ZFAS1 in pancreatic adenocarcinoma tissue samples and cell lines was further detected by quantitative real-time PCR and in situ hybridization. The functions of ZFAS1 in pancreatic adenocarcinoma in vitro and in vivo were investigated by further bioinformatic analysis. Dual-luciferase reporter assays were used to confirm the binding of ZFAS1/miR-3924 and miR-3924/ROCK2, and rescue assays were performed to further investigate the underlying mechanism.

**Results:**

ZFAS1 overexpression in pancreatic adenocarcinoma was predicted and experimentally verified. ZFAS1 silencing inhibited pancreatic adenocarcinoma metastasis in vitro and in vivo. The competing endogenous RNA mechanism of ZFAS1 was also identified.

**Conclusions:**

Our results demonstrated the promotive effect of ZFAS1 on pancreatic adenocarcinoma metastasis and suggested its potential role as a novel regulator of ROCK2.

## Background

Pancreatic adenocarcinoma (PAAD) is one of the most malignant tumours, with increasing mortality and a 5-year survival rate of less than 8% [1]. Considering that the current treatments for PAAD including surgery, chemotherapy and immunotherapy cannot obviously improve patient prognosis, more in-depth and broader studies exploring the molecular mechanism of PAAD progression are needed.

After the Human Genome Project was completed in 2003, more than 90% of long non-coding RNAs (lncRNAs), which are longer than 200 nucleotides (nts), were considered useless molecules because of their non-protein coding properties [2], but after a growing number of studies suggested that lncRNAs could regulate gene expression at the epigenetic and other levels [3, 4], scholars are paying more attention to the correlations between lncRNAs and cancers. The lncRNA ZFAS1, the antisense transcript of the gene ZNFX1, is abnormally expressed in many cancers. It was first found to be a tumour suppressor gene in breast cancer [5], but subsequent studies in other cancers reached the opposite conclusion [6, 7]. Although there are already studies on ZFAS1 in other cancers, few ZFAS1-related studies in PAAD have been published, and the expression level and underlying molecular mechanism of ZFAS1 in PAAD remain unknown.

In this study, microarray data were downloaded from the Gene Expression Omnibus (GEO) database to investigate the expression levels of ZFAS1 and other differentially expressed lncRNAs in PAAD. The expression level and clinical significance of ZFAS1 in PAAD were further investigated by analysing data from other databases and our microarray. Furthermore, we validated the function of ZFAS1 in PAAD and its underlying mechanism according to gene set enrichment analysis (GSEA) results, aiming to identify a novel potential therapeutic target for PAAD treatment.

## Methods

### Differential expression analysis of microarray data

Nine datasets (GSE14245, GSE15471, GSE21654, GSE27890, GSE32676, GSE42252, GSE46385, GSE51798, and GSE106189) based on the GPL570 Affymetrix Human Genome U133 Plus 2.0 Array Platform (Affymetrix, USA) were downloaded from the GEO database (http://www.ncbi.nlm.nih.gov/geo/), the raw data (CEL files) were normalized, and the background was adjusted by a robust multi-array average (RMA) algorithm and log_2_ transformed. Normalized data divided into tumour samples (n = 252) and normal samples (n = 65) were annotated by using the transcript ID and RefSeq ID supported by the GPL570 platform. Probes with no gene symbol or genes corresponding to several probes were removed or averaged. The limma package [8, 9] was used to find genes differentially expressed between the PAAD samples and normal samples. P-values were adjusted for a false discovery rate (FDR) using the Benjamini–Hochberg method (FDR < 0.05, |log_2 _(fold change) (FC)| > 0.585) [10]. The UALCAN (http://ualcan.path.uab.edu) [11], Gene Expression Profiling Interactive Analysis (GEPIA) (http://gepia.cancer-pku.cn) [12] and ONCOMINE databases (https://www.oncomine.org) were used to validate the expression level of ZFAS1 and its correlations with clinicopathological factors.

### Prediction of target miRNAs and mRNAs

DIANA-LncBase v2.0 (http://diana.imis.athena-innovation.gr/DianaTools/) and starBase v3.0 (http://starbase.sysu.edu.cn/) were used to predict lncRNA-binding microRNAs (miRNAs) [13, 14]. TargetScan 7.2 (http://www.targetscan.org/vert_72/) and miRDB (http://mirdb.org/) were used to predict mRNA targets [15, 16]. GSEA was conducted by using the GSEA tool (http://software.broadinstitute.org/GSEA/index.jsp). The Kyoto Encyclopedia of Genes and Genomes (KEGG) gene sets (c2.cp.kegg.v6.2.symbols) of the Molecular Signatures Database (MSigDB) were used to find genes associated with high ZFAS1 expression, and the results (normal p value < 0.05) were selected as enriched gene sets.

### Wound healing assay

Cells were seeded in a six-well plate and grown to confluence. Then, a line was scratched with a 1000-µl sterile pipette tip, and the medium in the wells was replaced with serum-free medium. The wound was imaged with an inverted microscope at an appropriate time. The results were analysed with Image J software.

### Western blot analysis

After 48 h of transfection, cells were harvested, and proteins were extracted. Then, a BCA protein assay kit was used to quantify the protein concentration, and proteins were separated on 10% (FAK and ROCK2) or 12% (RHOA) SDS-PAGE gels and transferred to PVDF membranes (Millipore, USA). Then, the membranes were blocked with 5% skim milk at 37 °C for one and a half hours. The primary antibodies were as follows: anti-ROCK2 (ab125025, Abcam, USA), anti-FAK (ab40794, Abcam, USA), anti-RHOA (ab187027, Abcam, USA) and anti-β-ACTIN (ab8227, Abcam, USA).

### Tissue microarrays

Tissue microarrays containing a total 170 samples including 28 cancer samples and 71 pairs of PAAD and noncancerous adjacent tissue samples were obtained from Shanghai Outdo Biotech (Shanghai, China). All follow-up information and clinical and pathological data were available. Among all 170 points, 18 points were excluded for dropping or uncertainty regarding the pathological type, and the remaining samples were all primary PAAD and normal tissue specimens. The expression level of ZFAS1 was evaluated independently by two people considering the signal intensity and percentage of ZFAS1-positive signals in an in situ hybridization (ISH) assay.

### Cell culture

Human PAAD cell lines SW1990, PANC1 and BXPC3 (Chinese Academy of Sciences Cell Bank, China), HEK-293T and human pancreatic ductal cell HPDE6C7 cell lines (Beina Culture Collection, China) were obtained. SW1990 (L-15, Gibco, USA), BXPC3 (RPMI 1640, HyClone, USA), PANC1, HPDE6C7 and HEK-293T (DMEM, HyClone, USA) were cultured with 10% foetal bovine serum (FBS; Corning, USA) at 37 °C with 5% CO_2_.

### RNA isolation and quantitative real-time PCR (qRT-PCR)

Total cellular RNA was extracted using TRIzol reagent (Invitrogen, USA) according to a standard protocol. cDNA production and qRT-PCR were performed by using the Reverse Transcriptase Kit (Takara, Japan) and SYBR Green Mixture (Takara, Japan) on the ABI 7500 System. Data were compared by using the 2-ΔΔCt method. The sequences were as follows: ZFAS1, forward, 5′-GCGAAAGCCATCTTTGGTTA-3′, reverse, 5′-GGGCAGGACAATAGCGTATG-3′; and GAPDH, forward, 5′-GGACCTGACCTGCCGTCTAG-3′, reverse, 5′-GTAGCCCAGGATGCCCTTGA-3′.

### RNA interference assay and lentiviral knockdown

The cell density was 70–80% before transfection with Lipofectamine 3000 (Invitrogen, USA). All the small interfering RNAs (siRNAs; GenePharma, China) were used at 20 nM, and the sequences were as follows: si-ZFAS-1, 5′-CCCTGTGCTTTCATGAAAGTGAAGA-3′, and si-NC,5′-CCAAAACCAGGCUUUGAUUGA-3′. A short hairpin RNA (shRNA) was used to silence ZFAS1 expression. Lentiviruses were stored at − 80 °C, and stable cells were selected with 1.5 µg/ml puromycin for 14 days. The sequences were as follows: sh-ZFAS1, 5′-CAGGAAGCCATTCGTTCTT-3′, and sh-NC, 5′-TTCTCCGAACGTGTCACGT-3′.

### ISH

ISH was performed by a conventional method with a few key reaction conditions. Samples were treated with 5 μg/ml Proteinase K (Takara, Japan) at 37 °C for five minutes, incubated with a probe (500 nM) at 50 °C for 1 h, and then washed with SSC (Sangon Biotech, China) at 50 °C for 20 min. An anti-DIG-AP reagent (Roche, Switzerland) was used at 4 °C for one night. NBT-BCIP (Roche, Switzerland) was used for colorimetric detection.

### Transwell migration assay

Approximately 5 × 10^4^ cells in 200 μl opti-DMEM were seeded in the upper chamber of a 24-well transwell system (Corning, USA), and the lower chamber was filled with 500 μl RPMI 1640 medium supplemented with 10% FBS. After being placed in a cell incubator for 24 h, the cells on the lower membrane surface were fixed with anhydrous alcohol and stained with crystal violet. Finally, the cells on the upper surface were removed with cotton buds.

### Dual-luciferase reporter assay

ZFAS1-wt/ZFAS1-mut and ROCK2-wt/ROCK2-mut were inserted into the pmirGLO reporter vector and then transfected with a miR-3924 mimic or miR-NC into cells with Lipofectamine 3000.After 48 h of transfection, luciferase activity was detected by using a dual-luciferase reporter assay kit (Promega, USA).

### In vivo metastatic model

Sh-NC/sh-ZFAS1 SW1990 cells (1 × 10^6^ cells/mouse) were injected into 4-week-old nude BALB/c mice (HFK Biotechnology, China) in two groups. The nude mice were sacrificed after 4 weeks, and tumours in the liver and lungs were observed after haematoxylin–eosin (HE) staining. This assay was approved by the Department of Laboratory Animal Science of China Medical University. The approval number was CMU2019201.

### Statistical analysis

Data are presented as the mean ± SD and were analysed with SPSS 24, GraphPad Prism 7, and MedCal 19. Differences were calculated by Student’s t-test. Survival curves were calculated by the Kaplan–Meier method and compared using the log-rank test. Fisher’s exact test was used to analyse the correlations between ZFAS1 expression and PAAD clinicopathological factors. P value less than 0.05 (*p < 0.05) was considered statistically significant [17].

## Results

### Prediction of ZFAS1 overexpression in PAAD by bioinformatic analysis

**Fig. 1:**
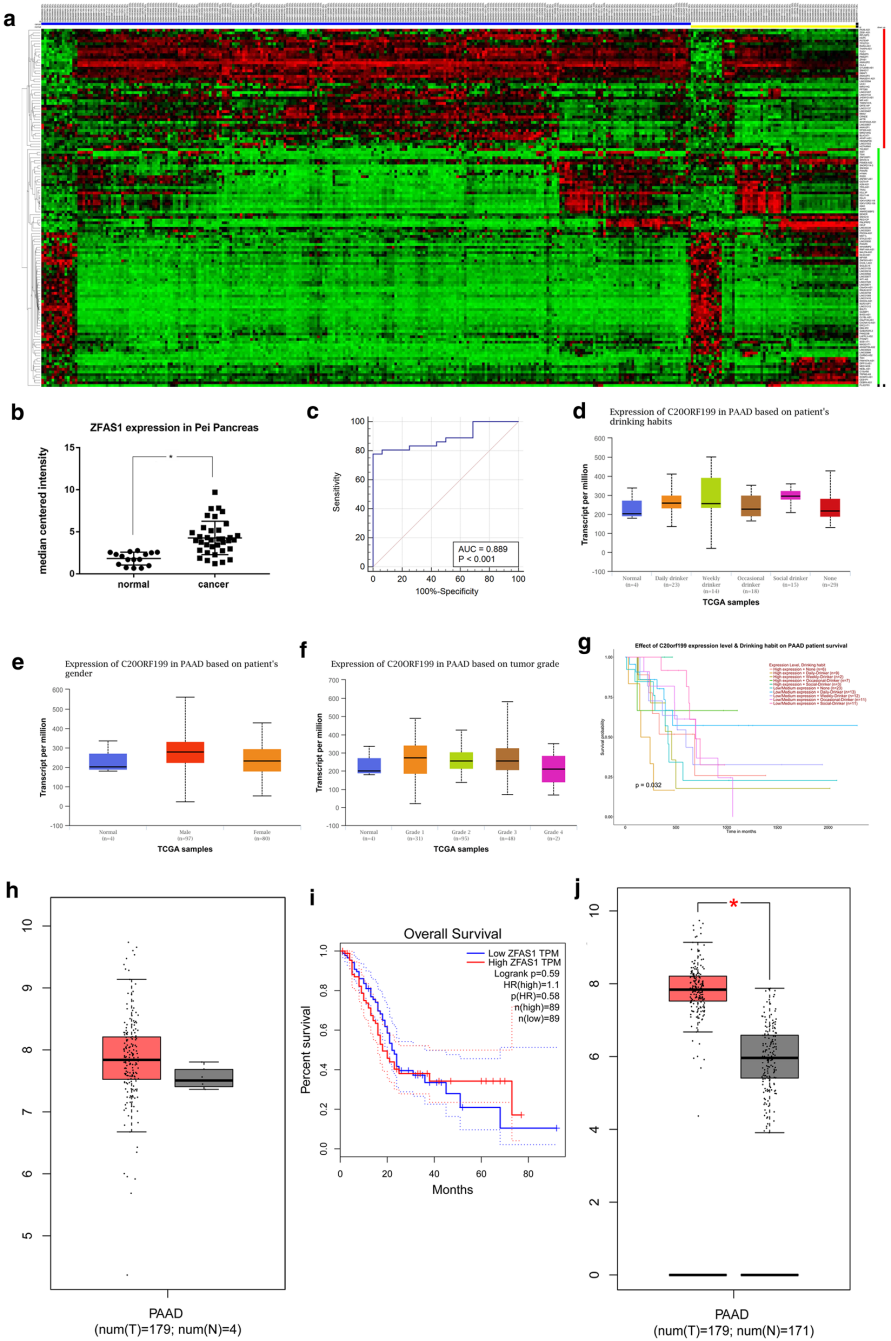
Prediction of ZFAS1 overexpression in PAAD by bioinformatic analysis. **a** All differentially expressed lncRNAs, including ZFAS1,in the merged GEO dataset are shown in the heat map. Data were normalized by the RMA algorithm and log_2 _transformed (blue: cancer samples; yellow: normal samples; red: upregulated lncRNAs; green: downregulated lncRNAs). **b** The ZFAS1 expression level in the Pei Pancreas dataset is shown, p < 0.05. **c** The ROC curve of the Pei Pancreas dataset showed an AUC for ZFAS1 of 0.889 (95% CI 0.771–0.959), p < 0.05. **d** ZFAS1 levels were positively associated with drinking habits (non-drinker vs occasional drinker, p < 0.05; occasional drinker vs weekly drinker, p < 0.05; weekly drinker vs daily drinker, p < 0.05). **e** Males had higher ZFAS1 levels than females, p < 0.05. **f** ZFAS1 levels were positively associated with tumour grade (grade 1 vs grade 3, p < 0.05). **g** Patients with different ZFAS1 levels and drinking habits showed survival differences, p < 0.05. **h**, **i** A TCGA-based analysis showed no difference in ZFAS1 expression or survival of patients stratified by ZFAS1 expression when comparing tumour tissue samples (n = 179) with normal tissue samples (n = 4). **j** The addition of normal samples from the GTEx database to the TCGA dataset (sample size increased from 4 to 171) improved the significance of the ZFAS1 expression difference, *p < 0.05. The vertical axes in **h**, **j** are log_2_(TPM + 1)

To investigate the differential expression of ZFAS1 and other lncRNAs in PAAD, nine GEO datasets were normalized and background adjusted to perform differential expression analysis. Forty-four lncRNAs, including ZFAS1, were identified to have upregulated expression in PAAD (Table 1) (Fig. 1a), and ZFAS1 expression levels in PAAD were further validated by data from the ONCOMINE, UALCAN and GEPIA databases. The results for the Pei 2009 microarray in ONCOMINE supported the GEO results calculated with 52 samples (16 normal and 36 cancer tissue specimens), and the area under the curve (AUC) value of the receiver operating characteristic (ROC) curve was 0.889 (Fig. 1b, c). UALCAN and GEPIA, which are both online tools based on The Cancer Genome Atlas (TCGA), showed correlations between the ZFAS1 expression level and patient sex, tumour grade and drinking habits (Fig. 1d–f). Patients also showed survival differences when considering the combined effect of the ZFAS1 level and drinking habits (Fig. 1g). However, no significant differences in ZFAS1 expression or patient survival with stratification based on ZFAS1 expression were found according to the TCGA-derived results alone (Fig. 1h, i). Notably, once the addition of GTEx data increased the sample size of the TCGA-PAAD normal tissue samples (from 4 to 171), the significance of the ZFAS1 expression level difference in PAAD was improved immediately (Fig. 1j).

**Table 1:** Up-regulated lncRNAs in the merged GEO dataset

Gene	LogFC	Adj.P.Val	Regulated
ZFAS1	1.292114407	3.79E−10	Up-regulated
ZEB1-AS1	0.751786689	3.16E−08	Up-regulated
UCA1	1.431842139	2.46E−08	Up-regulated
TUG1	0.819486239	3.85E−12	Up-regulated
TP53TG1	0.635576903	9.28E−08	Up-regulated
TMEM191A	0.623671728	2.56E−07	Up-regulated
THAP9-AS1	1.613537794	7.11E−21	Up-regulated
SNHG17	1.022943348	4.65E−12	Up-regulated
SH3PXD2A-AS1	0.740309382	1.56E−06	Up-regulated
RPL32P3	0.727465001	3.06E−08	Up-regulated
RNASEH1-AS1	0.61011974	3.68E−08	Up-regulated
RARA-AS1	0.639837801	7.40E−11	Up-regulated
PP7080	0.763093938	6.47E−06	Up-regulated
POTEKP	0.642481197	4.36E−06	Up-regulated
PMS2P3	0.632825563	1.18E−10	Up-regulated
PMS2P1	0.900767335	4.30E−13	Up-regulated
PAX8-AS1	0.897048546	1.77E−05	Up-regulated
OTUD6B-AS1	0.746209247	1.89E−11	Up-regulated
OR7E14P	1.188067838	3.01E−11	Up-regulated
NRAV	0.622272261	8.29E−13	Up-regulated
MIR31HG	0.691318092	5.08E−05	Up-regulated
MIR210HG	0.872969962	2.99E−08	Up-regulated
MIF-AS1	0.760101248	1.85E−09	Up-regulated
LINC01503	0.713069329	2.38E−07	Up-regulated
LINC01207	0.967896702	0.001919014	Up-regulated
LINC01137	0.615492033	8.09E−09	Up-regulated
LINC01133	0.855940052	1.57E−08	Up-regulated
LINC00857	0.852965802	3.37E−12	Up-regulated
LINC00467	0.771416443	7.23E−10	Up-regulated
LINC00094	0.780486158	1.15E−11	Up-regulated
JHDM1D-AS1	0.635281024	8.44E−11	Up-regulated
HOTAIRM1	0.617801402	0.001358296	Up-regulated
HMGN2P46	0.74496713	5.94E−08	Up-regulated
HLA-J	0.959015425	5.48E−07	Up-regulated
HCP5	1.086029552	1.96E−08	Up-regulated
GBAP1	0.892668634	1.67E−12	Up-regulated
EP300-AS1	0.988390142	1.40E−09	Up-regulated
CRNDE	1.991179914	1.82E−19	Up-regulated
BLACAT1	1.485832133	1.33E−14	Up-regulated
APTR	0.752779098	2.27E−15	Up-regulated
ANXA2P3	0.711844952	3.92E−16	Up-regulated
ANXA2P2	1.753775455	5.51E−21	Up-regulated
ANXA2P1	0.667336504	1.29E−10	Up-regulated
AFAP1-AS1	1.486798093	1.47E−13	Up-regulated

### Identification of the ZFAS1 expression level and clinical correlations in PAAD by experimental analysis

**Fig. 2:**
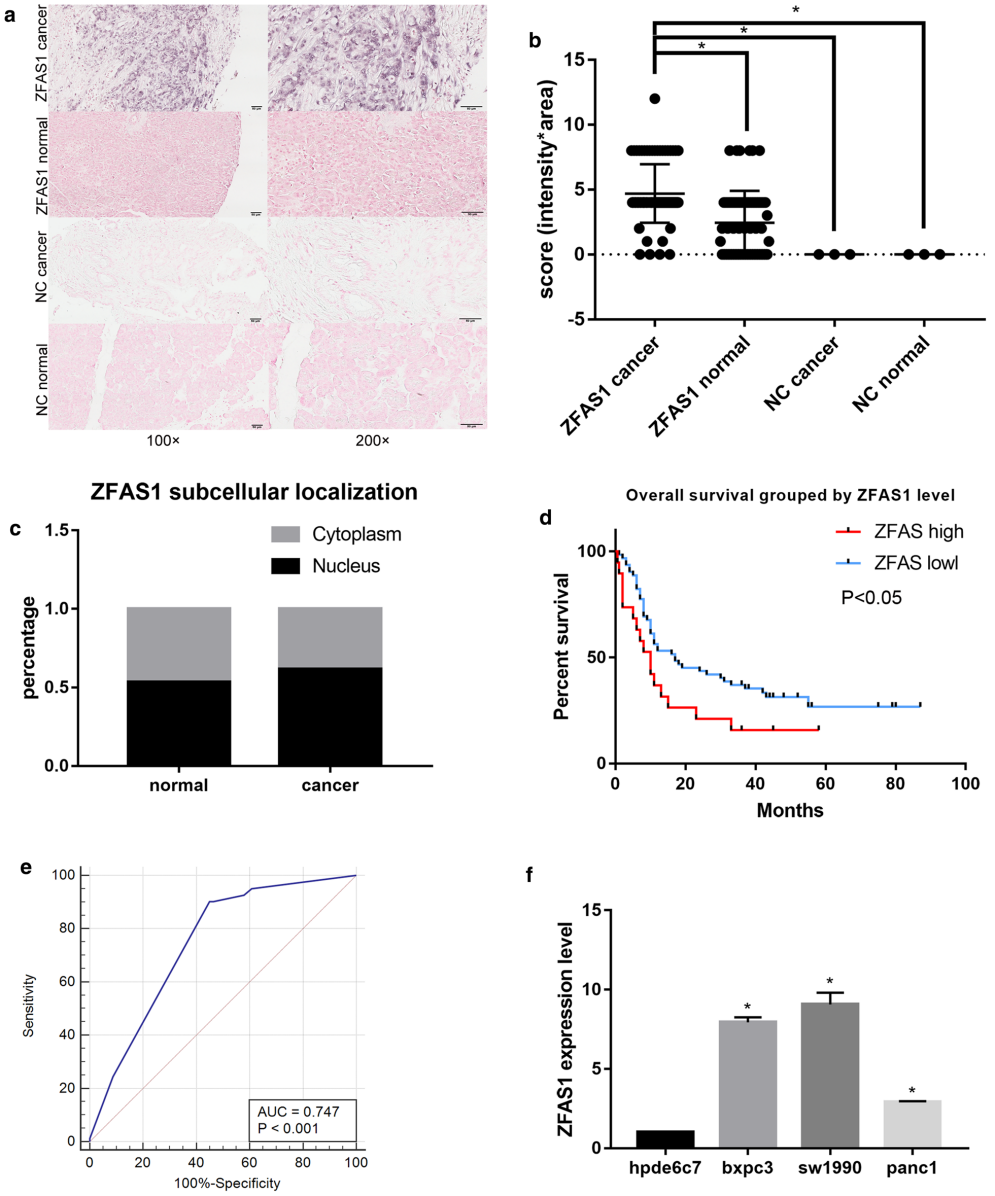
Identification of ZFAS1 overexpression and clinical correlations in PAAD by experimental analysis. **a** ZFAS1 expression in PAAD (n = 83) and normal tissue samples (n = 69) determined by ISH; scale bars, 50 μm. **b** Different ZFAS1 expression scores (intensity*area) in PAAD (n = 83) and normal tissue samples (n = 69) measured by ISH, p < 0.05. **c** ZFAS1 subcellular localization and expression level. **d** Kaplan–Meier survival analysis of PAAD patients stratified according to their ZFAS1 expression level, p < 0.05. **e** ROC curve showing the AUC for ZFAS1 (0.747, 95% CI 0.670–0.814), p < 0.05. **f** Relative ZFAS1 expression levels in PAAD cell lines and a human pancreatic ductal epithelial cell line measured by qRT-PCR (n = 3), *p < 0.05

A microarray with 170 points (71 paired and 28 single PAAD tissue samples) was obtained from Outdo Biotech (Shanghai, China). The expression level of ZFAS1 in the PAAD tissue specimens and its subcellular distribution were investigated by ISH, and the expression score was collected by calculating the product for intensity*area. The results showed that ZFAS1 was significantly more highly expressed in the cancer tissue samples than in the normal tissue samples and existed in both the cytoplasm and the nucleus (Fig. 2a–c). According to the ZFAS1 expression value, patients were divided into two groups. Furthermore, the patients with lower ZFAS1 levels had significantly longer overall survival times (Fig. 2d), and the AUC of the ROC curve was 0.747 (Fig. 2e). However, the ZFAS1 expression level showed no correlations with PAAD clinicopathological factors (Table 2). ZFAS1 expression in PAAD cell lines was also detected by qRT-PCR, and the results showed that ZFAS1 expression was also significantly upregulated in the PAAD cell lines (BXPC3, SW1990 and PANC1) compared with the normal cell line HPDE6C7 (Fig. 2f). Given that the SW1990 and BXPC3 cell lines had the highest ZFAS1 expression levels, they were selected for subsequent assays.

**Table 2:** Correlation between ZFAS1 expression and PAAD clinicopathological factors

Characteristics	N	ZFAS1 level		
		Low	High	
Total cases	83	63	20	
Gender	83			
Male		37	13	p = 0.794
Female		26	7	
Age(years)	82			
< 60		30	11	p = 0.798
≧60		32	9	
TNM stage	82			
I–II		61	19	p = 0.431
III–IV		1	1	
Tumor grade	18			
I		8	1	p = 0.576
III		6	3	
Tumor size(cm)	82			
< 5		41	16	p = 0.279
≧ 5		21	4	
Perineural invasion	36			
No		3	1	p = 1
Yes		23	9	
Lymph nodes	76			
Metastasis				
No		29	11	p = 0.285
Yes		30	6	

### ZFAS1 knockdown inhibits cell metastasis in PAAD

**Fig. 3:**
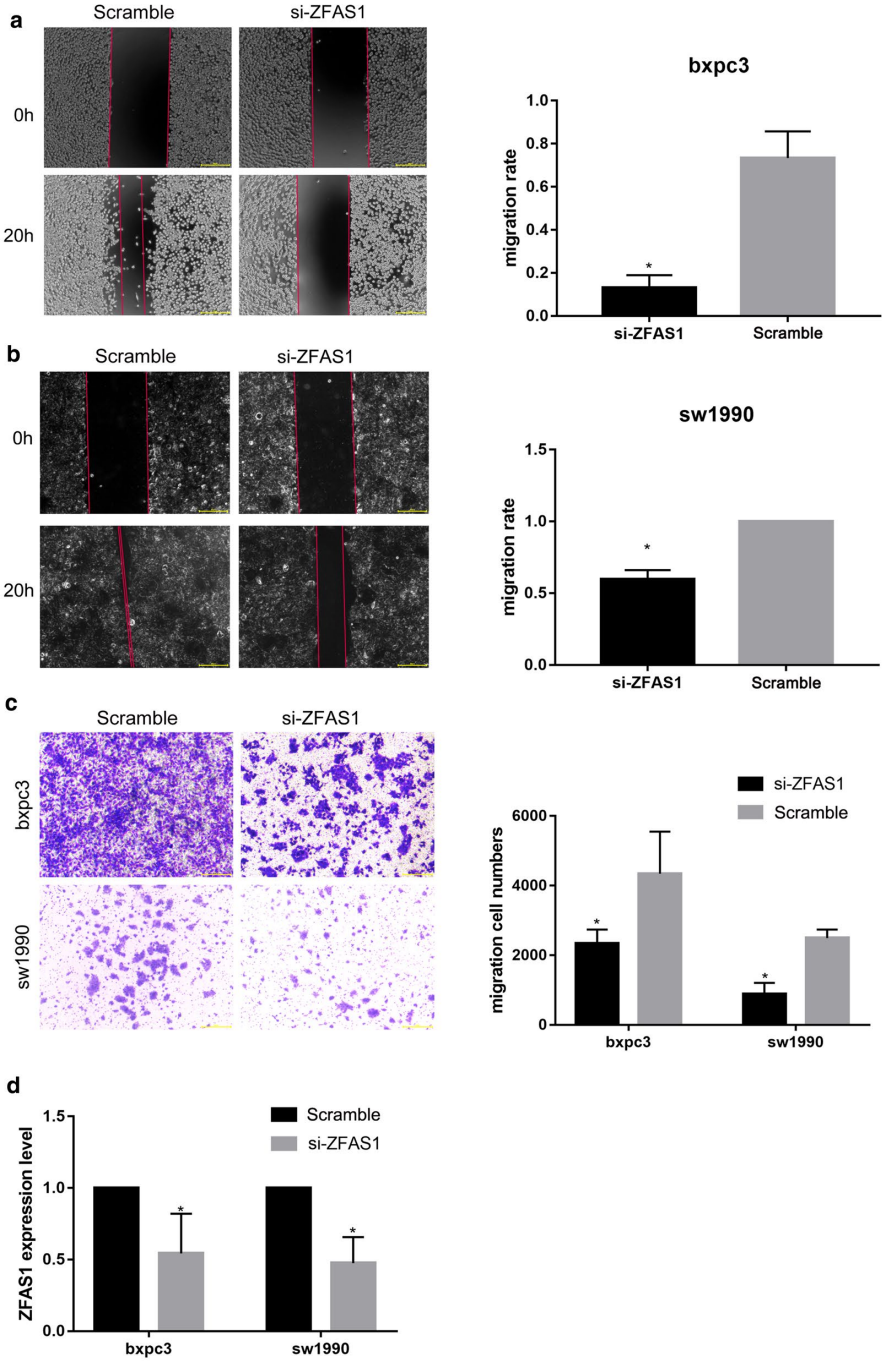
ZFAS1 knockdown inhibits cell metastasis in PAAD. **a**, **b** Wound healing assays showed that ZFAS1 knockdown inhibited the metastasis of BXPC3 and SW1990 cells (n = 3), *p < 0.05; scale bars, 50 μm. **c** Transwell migration assays showed that ZFAS1 knockdown inhibited the migration of BXPC3 and SW1990 cells (n = 3), *p < 0.05; scale bars, 50 μm. **d** Relative ZFAS1 expression in cells transfected with Scramble or si-ZFAS1 was measured by qRT-PCR (n = 3), *p < 0.05

To investigate the function of ZFAS1 in vitro, synthesized siRNAs were transfected into SW1990 and BXPC3 cells for 48 h. Wound healing (Fig. 3a, b) and transwell migration assays (Fig. 3c) showed that the metastasis of BXPC3 and SW1990 cells was inhibited after ZFAS1 knockdown. The transfection efficiency of si-ZFAS1 was detected with qRT-PCR (Fig. 3d). Thus, ZFAS1 knockdown was suggested to inhibit PAAD metastasis in vitro.

### ZFAS1 functions as a sponge of miR-3924

**Fig. 4:**
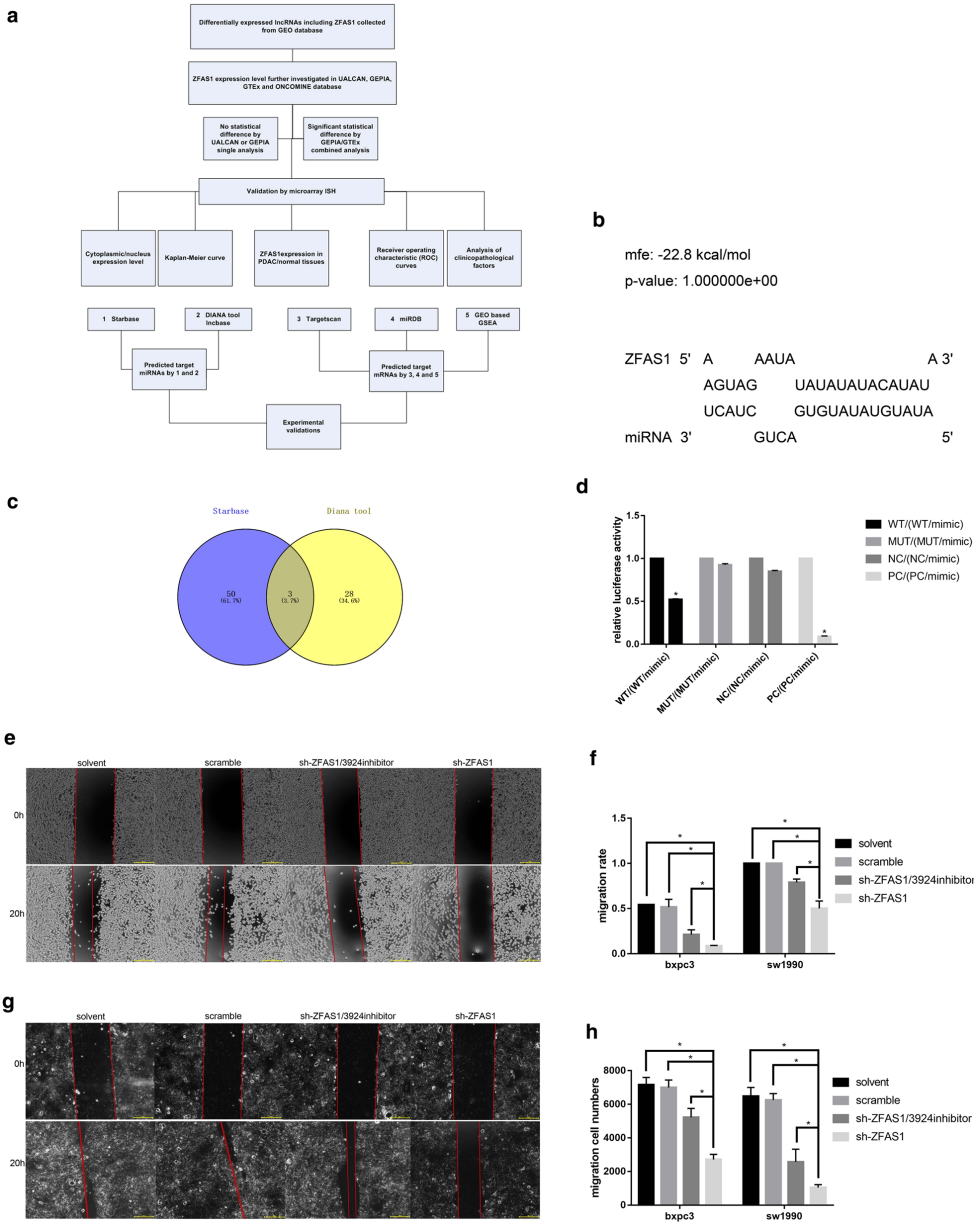

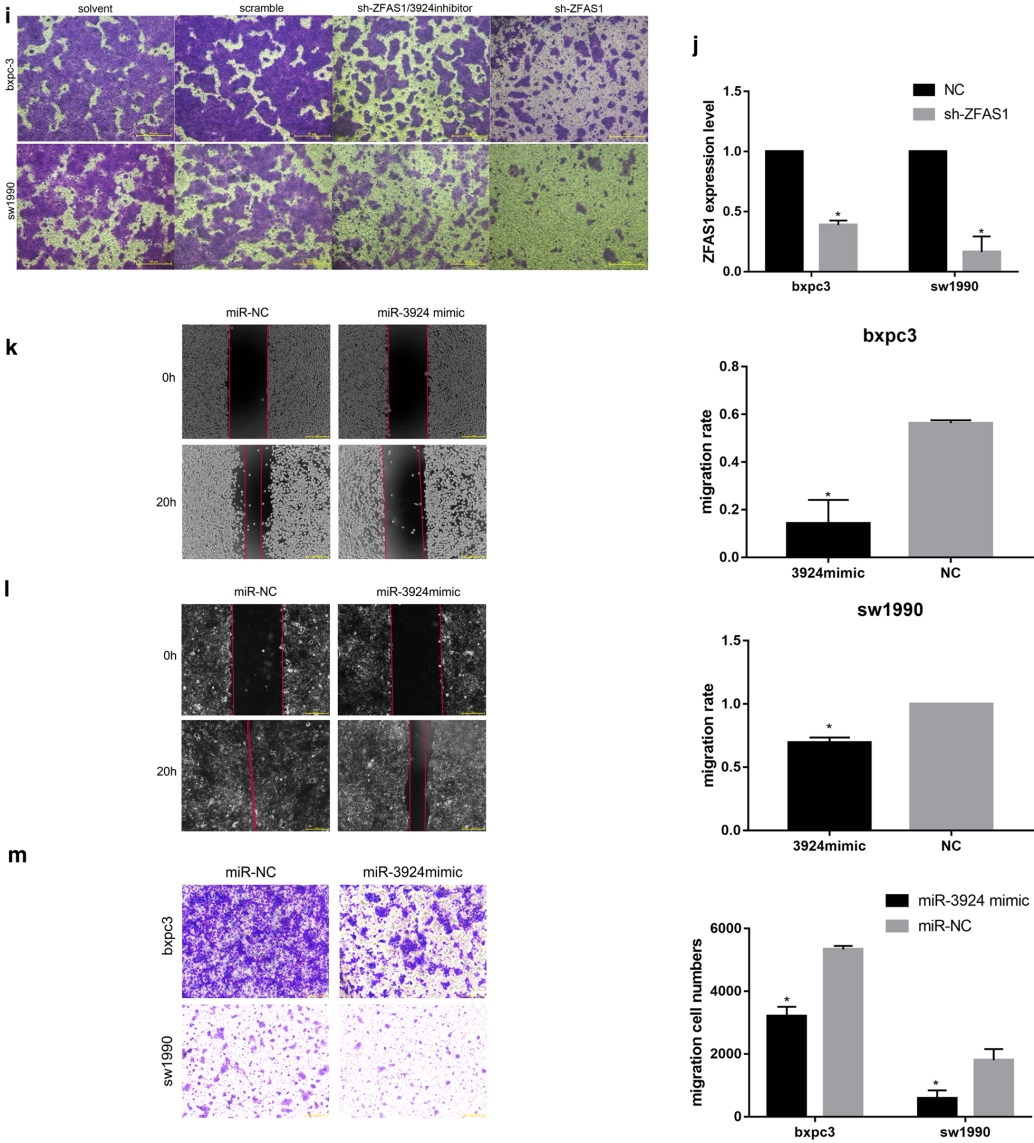
ZFAS1 functions as a sponge of miR-3924. **a** The flowchart of all bioinformatic analyses. **b** The binding site of ZFAS1/miR-3924 predicted by DIANA-LncBase 2.0. **c** Venn diagram of the target miRNAs predicted with starBase 3.0 and DIANA-LncBase 2.0. **d** HEK-293T cells transfected with ZFAS1 3′-UTR Wt, Mut, NC or PC reporter plasmids along with miR-3924 mimics. Relative luciferase activity was measured (n = 3), *p < 0.05. **e**–**g** Wound healing assays showing that miR-3924 inhibition reversed the effects of ZFAS1 silencing (n = 3), *p < 0.05; scale bars, 50 μm. **h**, **i** Transwell migration assays showing that miR-3924 inhibition reversed the effects caused by ZFAS1 silencing (n = 3), *p < 0.05; scale bars, 50 μm. **j** Relative ZFAS1 expression in cells transfected with sh-NC or sh-ZFAS1 was measured by qRT-PCR (n = 3), *p < 0.05. **k**, **l** Wound healing assays showing that miR-3924 overexpression inhibited the metastasis of BXPC3 and SW1990 cells (n = 3), *p < 0.05; scale bars, 50 μm. **m** Transwell migration assays showing that miR-3924 overexpression inhibited the migration of BXPC3 and SW1990 cells (n = 3), *p < 0.05; scale bars, 50 μm

The bioinformatic tools DIANA-LncBase, starBase and RNAhybrid were used to explore the target miRNAs of ZFAS1 (Fig. 4a). According to the intersection of the starBase and DIANA results, miR-3924 was chosen, and the binding site was predicted by RNAhybrid (Fig. 4b, c). Dual-luciferase reporter assay results showed that a miR-3924 mimic could inhibit the luciferase activity of ZFAS1-wt but not that of ZFAS1-mut (Fig. 4d). To further determine whether ZFAS1 plays its role through this interaction with miR-3924, we established BXPC3 and SW1990 cell lines with stable ZFAS1 silencing by using shRNA. After inhibiting miR-3924 expression in the stable ZFAS1-silenced BXPC3 and SW1990 cell lines, wound healing (Fig. 4e–g) and transwell migration assays (Fig. 4h, i) showed reversed results for both cell lines, indicating the antagonistic effects of ZFAS1 and miR-3924. The sh-ZFAS1 transfection efficiency was detected with qRT-PCR (Fig. 4j). In addition, because few miR-3924-related studies have been published, we also investigated the function of miR-3924 in PAAD cells, and the results showed that overexpressing miR-3924 could inhibit the metastasis of BXPC3 and SW1990 cells (Fig. 4k–m). Thus, ZFAS1 functions as a sponge of miR-3924.

### ROCK2 functions as the target of ZFAS1/miR-3924

**Fig. 5:**
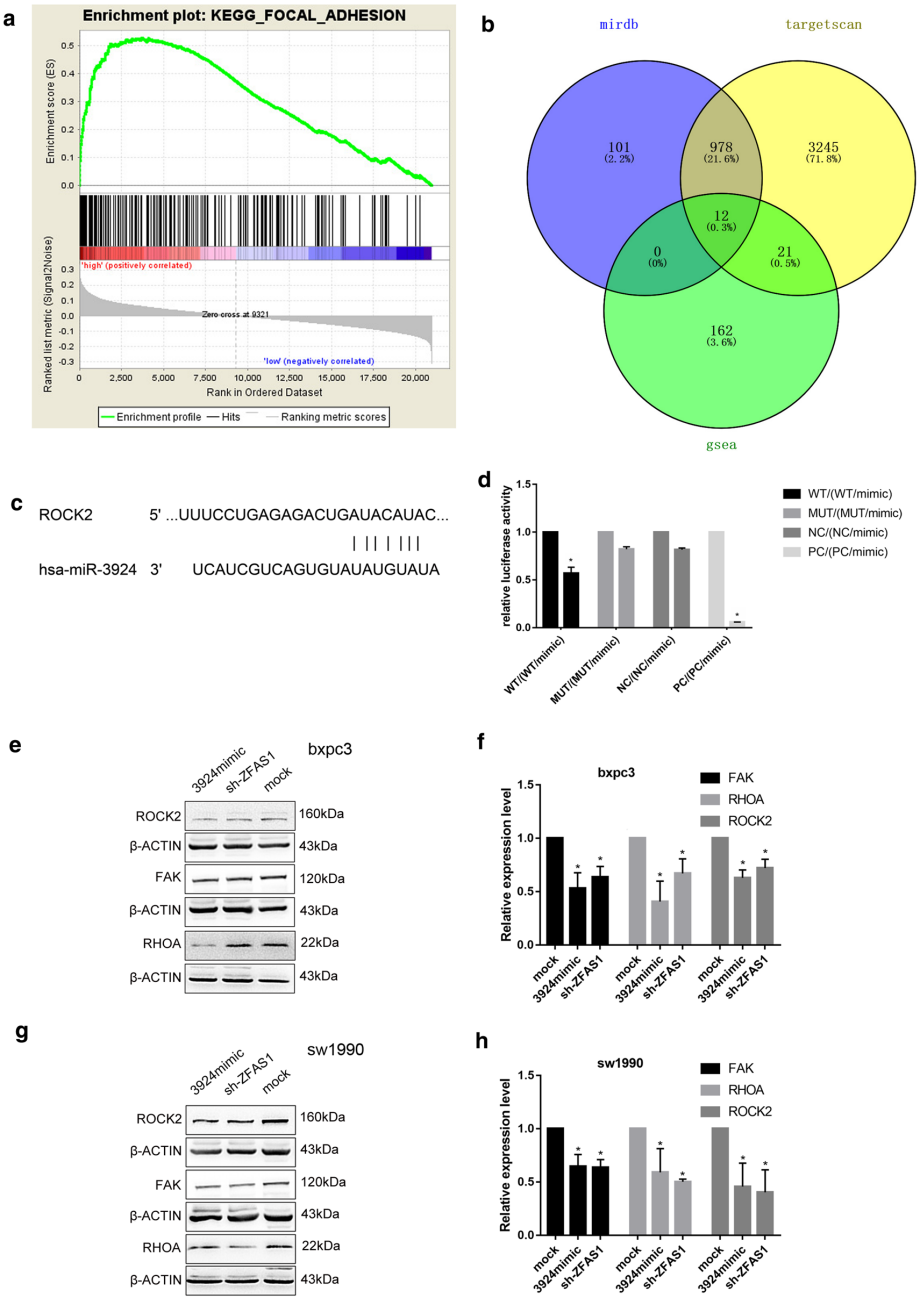
ROCK2 functions as the target of ZFAS1/miR-3924. **a** The differentially expressed genes in the high ZFAS1 expression group were focused primarily in the focal adhesion pathway. **b** The Venn diagram shows the target mRNAs predicted with GSEA, TargetScan 7.2 and miRDB. **c** The binding site of miR-3924/ROCK2 was predicted with TargetScan 7.2. **d** 293T cells were transfected with ROCK2 3′-UTR Wt, Mut, NC or PC reporter plasmids along with miR-3924 mimics, and relative luciferase activity was measured (n = 3), *p < 0.05. **e**–**h** Western blot assays showed that the expression levels of FAK, RHOA and ROCK2 in the sh-ZFAS1 and miR-3924 mimic groups were lower than those in the mock group (n = 3), *p < 0.05

GSEA was performed according to the merged GEO data, and the results showed that a high ZFAS1 expression level was most correlated with focal adhesion (Table 3)(Fig. 5a). By intersecting the GSEA results and predicted miR-3924 mRNA targets, ROCK2 was selected as the mRNA target (Fig. 5b, c). Dual-luciferase reporter assays showed that the luciferase activity of the ROCK2-wt/miR-3924 mimic group was significantly lower than that of the ROCK2-wt group; furthermore, no difference was found between the ROCK2-Mut group and the ROCK2-Mut/miR-3924 mimic group (Fig. 5d). Western blot results showed that the expression levels of FAK, RHOA and ROCK2 were decreased after ZFAS1 knockdown or miR-3924 overexpression (Fig. 5e–h). Thus, ROCK2 is suggested to function as the target of ZFAS1/miR-3924.

**Table 3:** ZFAS1 overexpression related pathways by GSEA

Name	Size	Es	Nes	Nom p-val
KEGG_FOCAL_ADHESION	194	0.53620225	1.7095456	0.004081633
KEGG_ECM_RECEPTOR_INTERACTION	81	0.5935364	1.644135	0.043737575
KEGG_MTOR_SIGNALING_PATHWAY	50	0.5283579	1.5339531	0.035196688

### ZFAS1 silencing inhibited tumour metastasis in vivo

**Fig. 6:**
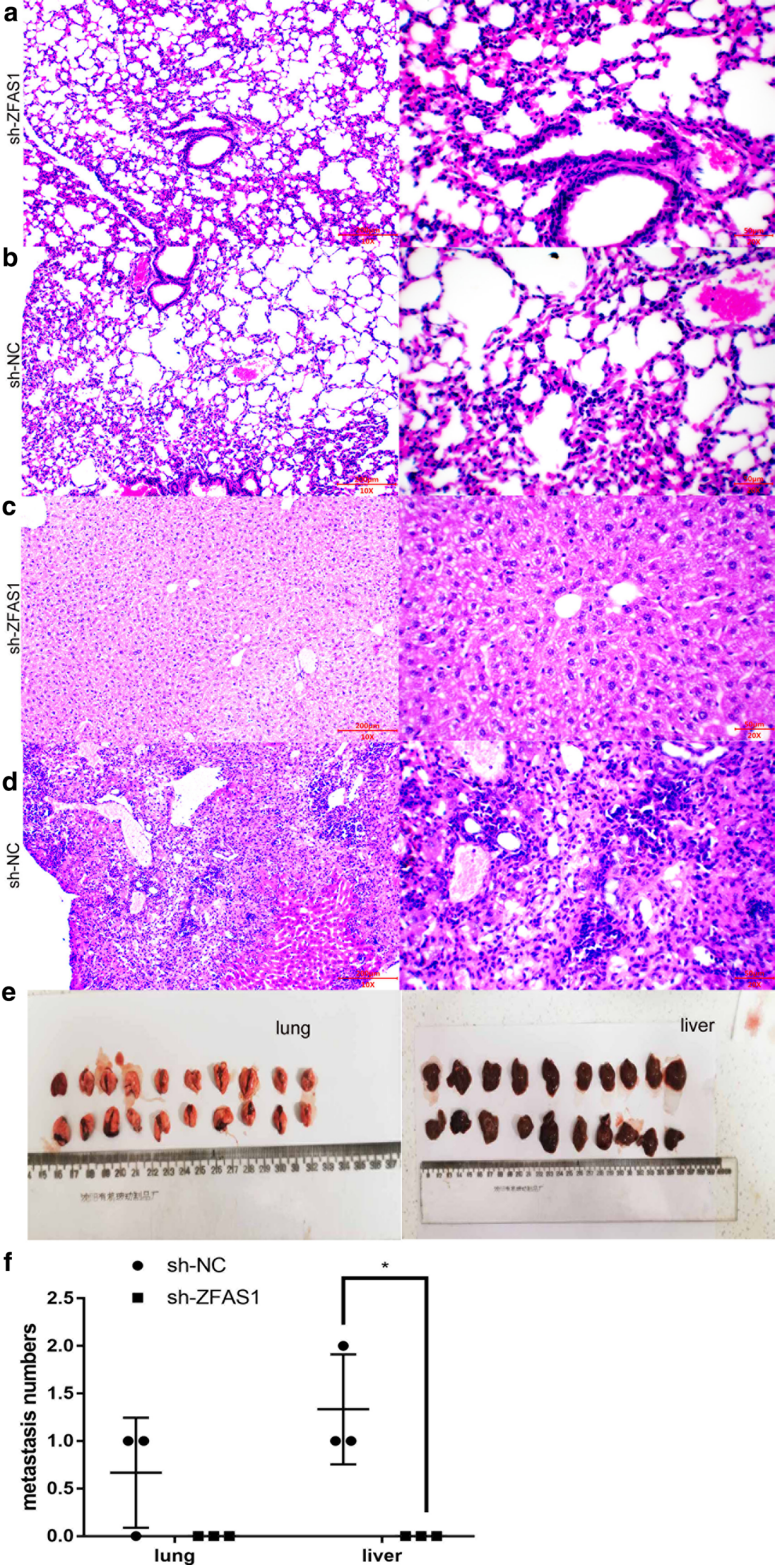
ZFAS1 silencing inhibited tumour metastasis in vivo. **a**–**d** Compared with sh-NC, sh-ZFAS1 did not inhibit metastasis to the lungs (**a**, **b**) but significantly inhibited metastasis to the liver (**c**, **d**); left: 100 × , scale bars: 200 μm; right: 200×, scale bars: 50 μm. **e** Nude mice were divided into two groups (sh-ZFAS1/sh-NC). Four weeks after tumour cell injection, the nude mice were sacrificed, and the livers and lungs of the mice in the two groups were photographed. **f** sh-ZFAS1 inhibited liver metastasis but not lung metastasis (n = 3), *p < 0.05

To investigate the biological function of ZFAS1 in vivo, stable sh-ZFAS1- and sh-NC-transfected SW1990 cells were selected with puromycin, and tumour metastasis models were established by intravenous injection. The results showed that compared with the NC group, the ZFAS1 silencing group exhibited significantly inhibited liver metastasis but not lung metastasis in vivo (Fig. 6).

## Discussion

Over the past two decades, while there have been rapid declines in the prostate cancer and breast cancer mortality rates, the incidence and mortality rates of PAAD have been growing, so study of the mechanisms underlying PAAD progression is urgently needed. Recently, considerable research has reported that lncRNAs play important regulatory roles in the developmental processes of tumours, including PAAD. For example, silencing lnc958 was shown to inhibit PAAD progression by inhibiting PAX-8 [18]. The downregulation of TUG1 expression was connected with the inhibition of tumour growth and invasion in PAAD [19]. HULC was also found to promote PAAD cell progression by downregulating miR-15a expression [20]. ZFAS1, as a novel star lncRNA candidate after HOTAIR and MALAT1, is abnormally expressed in various cancers; however, few ZFAS1-related studies on PAAD have been published.

To avoid results with low reliability caused by the small sample size and high false positive rate of a single chip, we performed differential expression analysis of PAAD by analysing a pool of nine datasets from the GEO database. ZFAS1 was found to be significantly overexpressed in PAAD. Bioinformatic data from ONCOMINE but not the TCGA supplemented the GEO results. Given the significant difference detected when the normal sample number in the TCGA-PAAD dataset was increased, the results of the single TCGA-PAAD-based bioinformatic analysis may require further consideration (Fig. 1h, j).

The overexpression of ZFAS1 in PAAD and its clinical correlations were further experimentally verified based on bioinformatic analysis. The UALCAN results showed that the ZFAS1 expression level was significantly correlated with PAAD grade, sex and drinking habits. In addition, focal adhesion, extracellular matrix receptor interactions and the mTOR signalling pathway were correlated with high ZFAS1 expression according to the GSEA results. Considering these metastasis-related pathways, ZFAS1 was suggested to regulate metastasis in PAAD. In line with this prediction, relative function assays demonstrated that ZFAS1 functions as an oncogene by regulating PAAD metastasis in vitro and in vivo.

Although lncRNAs do not encode proteins, they regulate biological processes at different levels in multiple ways. One of the major mechanisms is acting as competing endogenous RNAs (ceRNAs) by competitively binding target miRNAs and further regulating functional mRNA expression [21, 22]. The binding of ZFAS1/miR-3924 and miR-3924/ROCK2 was predicted by bioinformatic analysis and validated by dual-luciferase reporter assays. Additionally, miR-3924 showed the opposite effect to ZFAS1 in transwell and wound healing assays. In addition, miR-3924 inhibition could reverse the effect caused by ZFAS1 inhibition. Moreover, silencing ZFAS1 and overexpressing miR-3924 could both downregulate the expression levels of ROCK2, RHOA and FAK. Thus, ZFAS1 was suggested to promote PAAD metastasis via the RHOA/ROCK2 pathway by sponging miR-3924. It is worth noting that the expression levels of the focal adhesion proteins FAK, RHOA and ROCK2 in the SW1990 and BXPC3 cell lines were different (Fig. 5e, g). SW1990 cells showed significantly higher focal adhesion protein levels and a significantly higher migration rate in wound healing assays than BXPC3 cells. Whether correlations between focal adhesion protein levels and migration exist in PAAD needs further study, but if these correlations exist, they could be novel evidence indicating that focal adhesion pathway proteins promote metastasis in PAAD.

ROCKs are major effectors of the small GTPase RHOA, and the important roles of ROCK in cancer progression, including in cell metastasis, have been demonstrated by thousands of studies [23–25]. Moreover, ROCK inhibitors such as Y-27632 [26] and fasudil [27] have also been widely studied in various cancer cell lines and animal models and have shown significant benefits when used alone [28–30] or in combination with other chemotherapeutic drugs [31–33]. Although the role of ROCK2 shown in basic studies has not been questioned, only one clinical trial using the ROCK2 inhibitor AT13148 for cancer treatment was reported in 2012 (ClinicalTrials.gov identifier NCT01585701). Barriers that prevent the known ROCK signalling knowledge from being translated into anticancer treatments exist; considering the promotive effect of ZFAS1 on ROCK2 via the identified ceRNA mechanism, this promotion may provide a novel solution for the clinical dilemma of ROCK2 inhibitors.

Considering that ZFAS1 still showed a degree of expression in normal pancreatic tissue and the effects on the ZFAS1 expression level caused by multiple factors (sex, tumour grade and drinking habits), ZFAS1 may not be valuable for the diagnosis of PAAD currently. Pancreatic intraepithelial neoplasia (PanIN) and chronic pancreatitis samples should be collected to analyse the ZFAS1 expression level difference between cancer and precancerous lesions, so the diagnostic value of ZFAS1 in PAAD can be further validated.

## Conclusions

In summary, our data demonstrated the overexpression of ZFAS1 in PAAD and its clinical correlations with tumour grade and prognosis. Its promotion of cell metastasis mediated by regulating the miR-3924/ROCK2 axis was also demonstrated. We hope our study will provide new ideas for solving pancreatic cancer problems and translating ZFAS1/miR-3924/ROCK2 signalling knowledge into anticancer therapies.

## Data Availability

All GEO datasets (GSE14245, GSE15471, GSE21654, GSE27890, GSE32676, GSE42252, GSE46385, GSE51798, and GSE106189) and the ONCOMINE dataset (Pei Pancreas) are available from the GEO and ONCOMINE databases. The TCGA-PAAD results are available from the UALCAN and GEPIA databases. The pancreatic cancer microarray HPan-Ade170Sur-01 and relevant patient clinical information are available from the corresponding author by request. All original images and data for RT-PCR assays, Western blot analyses, ISH studies, transwell migration assays, wound healing assays, dual-luciferase reporter assays and our nude mouse metastasis model are available from the corresponding author by request.

## Abbreviations

PAAD: Pancreatic adenocarcinoma
lncRNAs: Long non-coding RNAs
GEO: Gene Expression Omnibus
GSEA: Gene set enrichment analysis
RMA: Robust multi-array average
FDR: False discovery rate
MSigDB: Molecular Signatures Database
ceRNAs: Competing endogenous RNAs
ROCKs: Rho-associated coiled-coil kinases
PanIN: Pancreatic intraepithelial neoplasia
